# Blends of Thermoplastic Polyurethane and Polydimethylsiloxane Rubber: Assessment of Biocompatibility and Suture Holding Strength of Membranes

**DOI:** 10.1155/2013/240631

**Published:** 2013-12-18

**Authors:** Krishna Prasad Rajan, Ahmed Al-Ghamdi, Ramesh Parameswar, G. B. Nando

**Affiliations:** ^1^Department of Chemical Engineering Technology, Yanbu Industrial College, Royal Commission for Jubail and Yanbu, P.O. Box 30436, Yanbu Industrial City 21 477, Saudi Arabia; ^2^Biomedical Technology Wing, Sree Chitra Tirunal Institute for Medical Sciences and Technology, Poojapura, Thiruvananthapuram, Kerala 695 012, India; ^3^Rubber Technology Centre, Indian Institute of Technology, Kharagpur 721302, India

## Abstract

In the present investigation, a compatibilized blend of thermoplastic polyurethane (TPU) and polydimethylsiloxane (PDMS) is prepared by using copolymer of ethylene and methyl acrylate (EMA) as a reactive compatibilizer. Detailed *in vitro* biocompatibility studies were carried out for this compatibilized blend and the material was found noncytotoxic towards L929 mouse fibroblast subcutaneous connective tissue cell line. Microporosity was created on the surface of membranes prepared from the blend material by adopting the crazing mechanism. Cell proliferation and growth studies on the membranes surface showed that the microporous surface favoured ingrowth of the cells compared with a nonmicroporous surface. Suture holding strength studies indicate that the microporous membranes have enough strength to withstand the cutting and tearing forces through the suture hole. This blend material could be evaluated further to find its suitability in various implant applications.

## 1. Introduction

Thermoplastic polyurethane (TPU) and polydimethylsiloxane rubber (PDMS) are two well known biomaterials with excellent biocompatibility and biostability. A large volume of literature resources are available describing the biomedical applications of these two materials [1–6]. If TPU and PDMS are blended together, the mechanical strength and biocompatibility of TPU can be added to the inertness and biocompatibility of PDMS. The resulting blend material has several advantages. One of the main advantages is the formation of a thermoplastic elastomer material, which has the properties of an elastomer at room temperature and allows processing in conventional plastic processing equipments like injection moulding machine or an extruder. Furthermore, it allows tailoring of the end use property requirements by adjusting the ratio of the blend components. Being a thermoplastic elastomer material, the need for crosslinking the PDMS component can be avoided, which is a major gain. This is because crosslinking involves the use of various curing chemicals and there is a chance for migration of these chemicals to the surrounding tissues during the long term implantation period of the material, which will result in toxicity for the surrounding tissues and results in the rejection of the implant from the body [7].

The main obstacle in blending TPU with PDMS is the formation of an immiscible blend which will lead to phase separation. This can be addressed by adopting the reactive blending or in situ compatibilization technique using a copolymer capable of making specific interactions with the blend components. The authors have reported the in situ compatibilization of an 80 : 20 blend of TPU and PDMS compatibilized with a copolymer of ethylene and methyl acrylate (EMA) [8]. A compatibilized blend of TPU and PDMS could be used for a variety of biomedical applications where the biocompatibility, strength, and toughness of TPU and the inertness, biocompatibility, and flexibility of PDMS are required. Such applications include artificial organs, scaffolds for tissue engineering, membranes for guided tissue regeneration, soft tissue replacements, cartilage replacements, and so forth.

Microporous structures are generally required for biomaterials intended for implantation. The theory behind the microporous implant is that a controlled network of porosity will improve the invasion and proliferation of cells on to the biomaterial surface [10–12]. There are different methods for the development of microporous structures in implants. Miyamoto et al. [13] described a technique to create microporosity in small-caliber vascular prostheses in which calcium carbonate (mean particle size, 8 *μ*m) was incorporated with polyurethane during the fabrication of the prosthesis, followed by placing the tube in hydrochloric acid to remove the calcium carbonate and thereby created microporosity on the device.

In the present study, microporous structures of compatibilized blends of TPU and PDMS were formed by a crazing mechanism proposed by Chandavasu et al. [14, 15]. In the blend system, the minor phase that is well dispersed in the matrix, acts as a stress concentrator. The porosity is introduced by drawing the samples. When the sample is deformed by drawing, the minor phase, domains are debonded due to the weak adhesion between phases. Microcracks are initiated at points of high stress concentration which are at interface between the two phases. Subsequent growth occurs by a process in which crazes propagate into the major phase of blends. Shear yielding also occurs along with the crazing. Rates of craze initiation and growth depend upon the rate of applied stress [16].

One of the major problems associated with synthetic membranes for biomedical applications is the cutting or tearing of the membrane through the suture hole. Suture holding strength or suture tearing strength is a measure of the mechanical resistance to cutting with a suture. This measurement is important for membranes intended to be tightly sutured with host tissues or organs.

The objectives of present study include the preparation of a compatibilized blend of TPU and PDMS and generation of microporosity on the blend surface, study the suture holding strength of the membranes, and carry out detailed evaluation of *in vitro* cytotoxicity of the blend.

## 2. Materials and Methods

### 2.1. Materials

Thermoplastic polyurethane (Desmopan KU 2-8600E, an ether type TPU, with melting point 190°C and specific gravity 1.11) was supplied by Bayer, India. PDMS (KE 151 U, with specific gravity 1.15) was obtained from Shin-Etsu, Japan. EMA (melting point 81°C and specific gravity 0.94) was supplied by Exxon, India.

### 2.2. Blend Preparation

Blends of TPU and PDMS with the various dosage of compatibilizer (0 to 10 wt%) were prepared using Haake Polylab System Rheomix 600P with cam rotors. The temperature for mixing was 190°C and rotor speed of 80 rpm was employed. Mixing time of 10 and 14 minutes were given for virgin TPU and the blends, respectively. The material after mixing was quickly transferred to a laboratory two roll mill (150 mm × 300 mm) and sheeted out at room temperature. From the blends, sheets of dimension 120 mm × 120 mm × 2 mm were obtained by compression molding in a heated press equipped with a water cooling system. Samples for various physical property tests were cut from these sheets using a specimen punching machine. Thin films were prepared by compression molding the blend samples between two parallel plates in the compression molding press. The samples were conditioned at 23°C for 24 hours before every test.

### 2.3. Development of Microporous Structure

The treatments used for the preparation of microporous structure include the following interrelated steps:

1. Blend samples in the form of rectangular strips were uniaxially drawn (100–600%) with respect to the original dimension to induce debonded interphase crazing. The growth of crazes is controlled by the degree of applied stress.
2. Samples were then held for 10 minutes at various temperatures (20–60°C) in the stretched condition to stabilize the microporous structure.

Universal Testing Machine (UTM) fitted with an environmental chamber (Hounsfield H10KS) was used to carry out the stretching experiments. The microporous structure was then observed through scanning electron microscope (SEM, S-2400 HITACHI, Japan).

### 2.4. Suture Holding Strength Measurement

**Figure 1:**
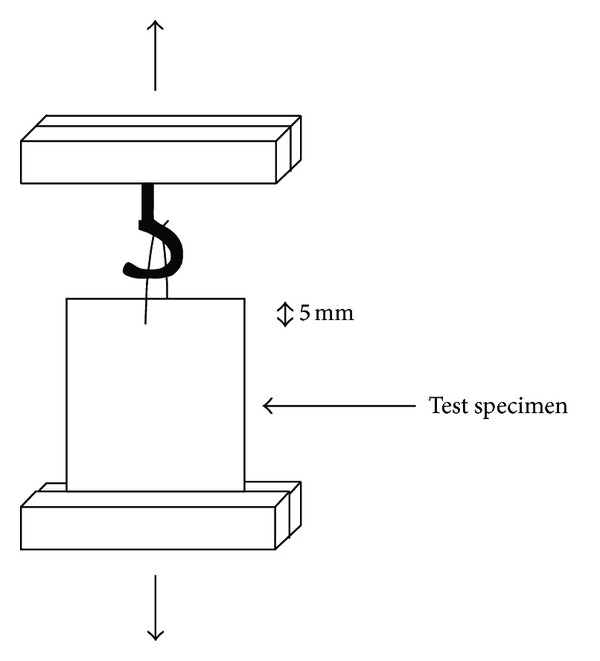
Measuring method for suture holding strength adopted from Reference [9].

The suture holding strength was measured using the one-point suspension method described by Matsumoto et al., [9] using a Universal Testing Machine. The sample was fixed with a stitch, made up of nylon suture placed 5 mm from one edge, and then the opposite edge was fastened to the grip of the UTM. A metallic hook was fixed on the other grip of the UTM and the suture was carefully held with the hook. A schematic of this arrangement is given in Figure 1. The cross-head speed given was 5 mm/min. The maximum stretching strength was measured and expressed as tensile force per 1 mm width of the membrane.

### 2.5. Biological Evaluation

#### 2.5.1. *In Vitro* Cell Culture Cytotoxicity

*In vitro* cytotoxicity study (direct contact) was conducted in the compatibilized blend samples. Button shaped samples were cleaned in an ultrasonic bath and subjected to *γ*-ray irradiation (2.5 Mrad) and used for the study. The *in vitro* cytotoxicity was assessed as per ISO-10993-5 (2002) using L929 mouse fibroblast subcutaneous connective tissue cell line procured from National Centre for Cell Sciences, Pune, India. The cells were maintained in RPMI 1640 medium (Himedia, Pune, India) supplemented with 10% foetal bovine serum (Sigma, USA) and 100 IU/mL penicillin and 100 *μ*g/mL streptomycin (medical grade). The culture was incubated at 37 ± 2°C in a humidified atmosphere containing 5% carbon dioxide with a medium change at an interval of 3 days.

#### 2.5.2. Direct Contact Test

Cytotoxicity of compatibilized blend samples by direct contact method was evaluated as per ISO-10993-5: 8.3 (2002). The control samples and the blend sample were placed in contact with the cells and incubated at 37 ± 2°C for 24 hours. High density polyethylene (HDPE) was taken as the negative control and organotin stabilized polyvinyl chloride (o-PVC) was taken as the positive control.

#### 2.5.3. Testing on an Extract

Cytotoxicity of compatibilized blend samples was also evaluated by testing on an extract as per ISO-10993-5 8.2 (2002). Material extract was prepared by incubating 0.1 g of the blend sample in the culture medium at 37 ± 2°C for 24 h. Phenol and tissue culture grade polystyrene (TCPS) were used as the positive and negative controls, respectively. To analyse cytotoxicity, culture medium from confluent cells were replaced with material extracts and cytotoxicity was assessed qualitatively after 24 h.

#### 2.5.4. *In Vitro* Cell Adhesion Studies

*In vitro* cell adhesion studies were performed using L929 cells for 48 ± 1 hours on blend samples before and after creating microporosity on the surface. Uniform number of cells were seeded on the samples and incubated at 37 ± 1°C under humidified atmosphere in presence of 5% CO_2_. After incubating for 2 days, the samples were washed with PBS and fixed with 2.5% glutaraldehyde in 0.1 m phosphate buffer for 30 min at 4°C. After the fixation, the samples were washed twice with phosphate buffer and then dehydrated in ethanol solutions (50% to 100%). The samples were kept in 100% ethanol until being subjected to critical point drying (CPD) to avoid water contamination. The samples were then sputter coated by gold and examined by scanning electron microscope (SEM, S-2400 HITACHI, Japan).

## 3. Results and Discussions

### 3.1. Compatibilization of the Blend

Based on the results obtained from torque rheometry (apparent viscosity values), mechanical property evaluation, infra red spectra, scanning electron microscopy (SEM), and atomic force microscopy (AFM), it was concluded that the optimum level of compatibilizer required to effectively compatibilize a polyblend system of 80 : 20 TPU and PDMS was 2 wt% of EMA. This was reported in our earlier publication [8]. The optimized blend system is used for generation of microporosity and biological evaluation.

### 3.2. Microporosity

**Figure 2:**
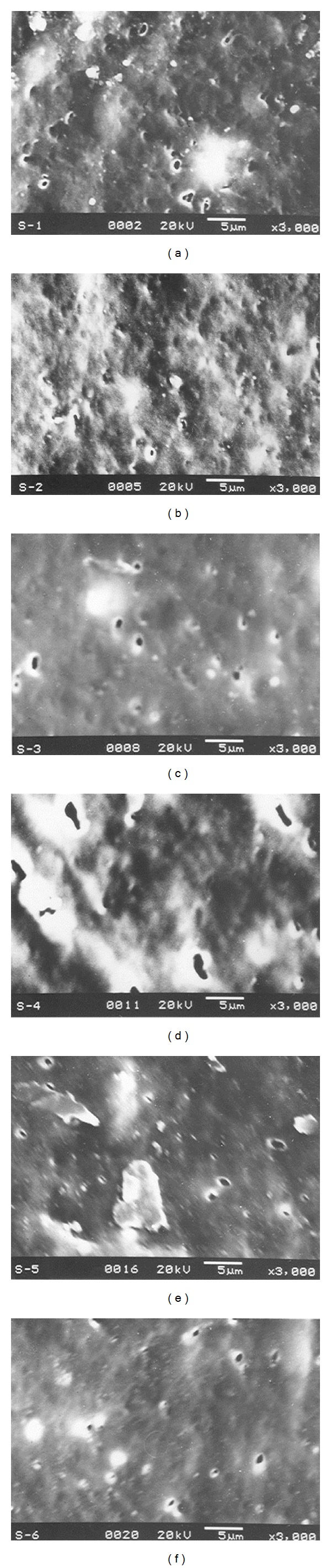
Scanning electron micrographs showing the various stages of experiments for optimization of microporosity on the surface of compatibilized blend.

Stretching experiments were carried out at various stretching speeds (200 mm/min–500 mm/min), to various extension (100–600%) at different temperatures (20–80°C) followed by holding the structure for 10 min in the stretched condition for stabilizing the morphology. Series of experiments were carried out to optimize the conditions such as optimum stretching speed, optimum percentage extension, and optimum temperature leading to the development of a more uniform microporous structure.  Figure 2 shows the scanning electron micrographs of various stages of this optimization experiments.

**Figure 3:**
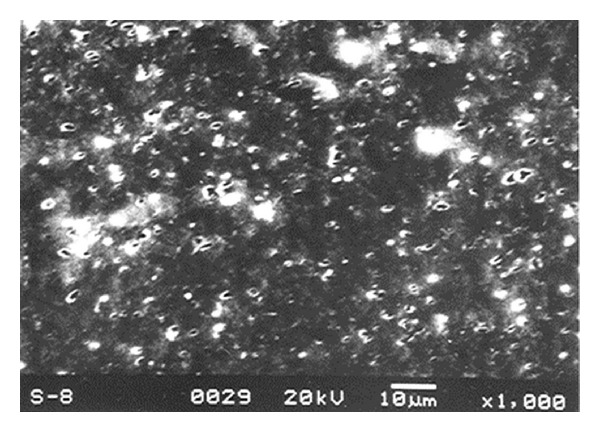
SEM micrograph of distribution of micropores in the compatibilized blend, stretched at optimum conditions.

It was observed that the stretching speed of 500 mm/minute, extension of 300%, and temperature of 60°C resulted in the optimum number of pores uniformly distributed over the blend surface. The scanning electron micrograph showing the microporosity in the compatibilized blend under these optimized conditions is shown in Figure 3. Large numbers of pores with size in the range 1–5 microns are visible on the surface. Also, the pores are distributed uniformly over the surface.

The contact angle measurements of these membranes are expected to provide more information regarding the wettability of the surfaces and thereby help to assess the hydrophilicity/hydrophobicity of the material, which are important parameters in controlling the cell proliferation and growth over the membrane.

### 3.3. Suture Holding Strength Measurement

The suture holding strength measurements were carried out on films of 80 : 20 blend of TPU : PDMS containing compatibilizer varying from 0 to 5 pbw (Table 1). The experiment was repeated after creating microporosity on the surface of the films under the optimum conditions. Suture holding strength and tearing resistance are found to be higher for the blend containing 2 pbw of compatibilizer.

### 3.4. *In Vitro* Cytotoxicity Studies

**Figure 4:**
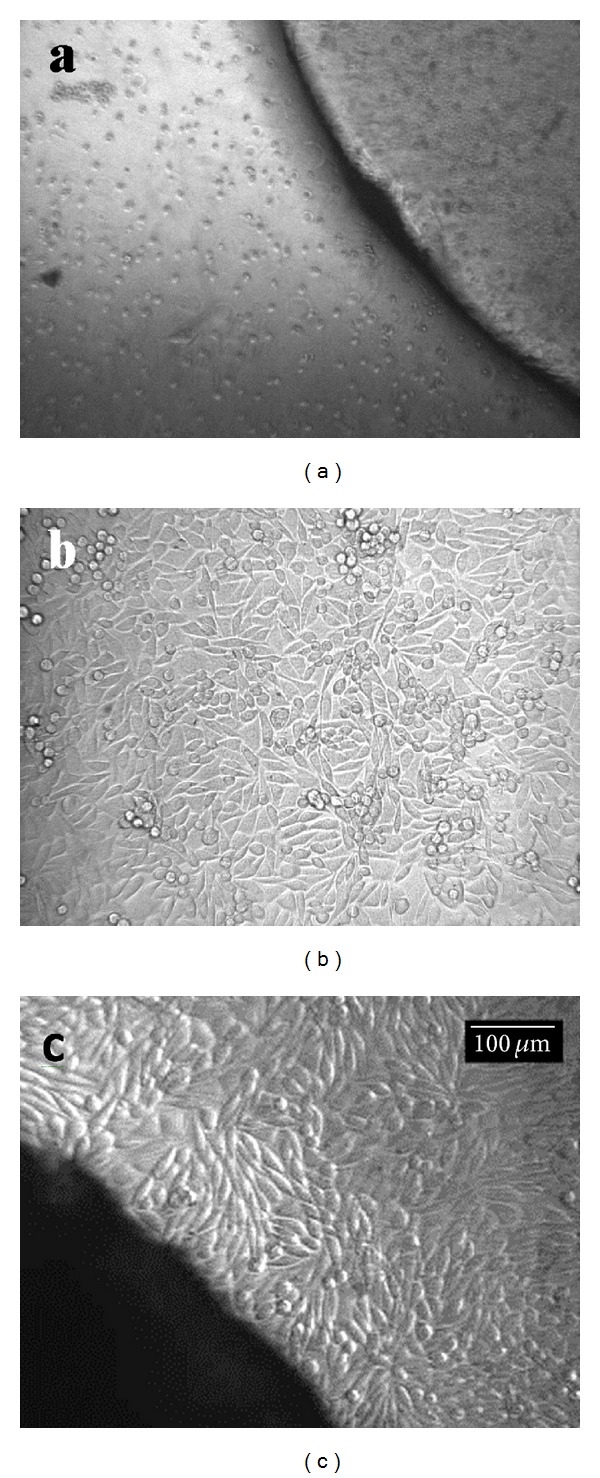
L929 cells incubated with (a) positive control, (b) negative control, and (c) 50 : 50 blend of TPU : PDMS containing 5 pbw of compatibilizer over 24 h.

**Figure 5:**
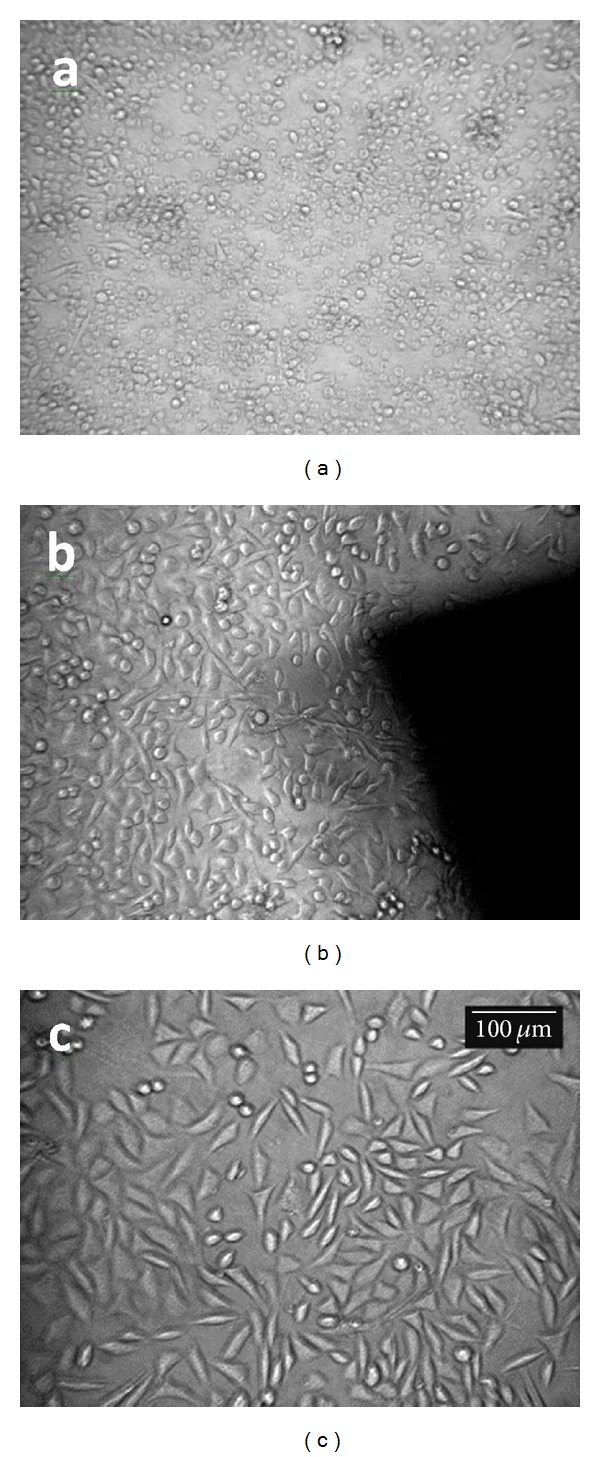
L929 cells incubated with (a) positive control, (b) negative control, and (c) 50 : 50 blend of TPU : PDMS containing 5 pbw of compatibilizer over 24 h.

**Figure 6:**
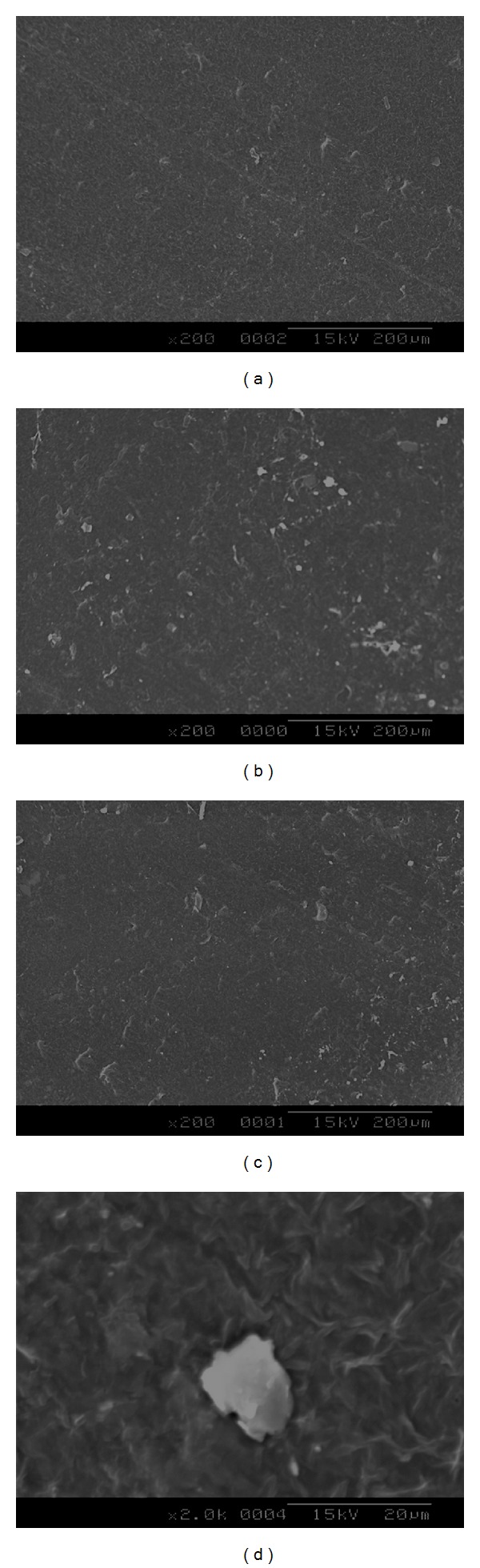
SEM micrographs showing the cell growth over the surface of blend samples without microporosity on surface.

*In vitro* cytotoxicity studies carried out on compatibilized blend showed that the blend material is noncytotoxic to L929 cells in culture. L929 mouse fibroblast cells are well proven cell lines for cytotoxicity evaluation of biomaterials [17]. In the direct contact study, the cells in contact with the blend material showed spindle morphology characteristic of mouse fibroblast cell line. Similar results were obtained for the test performed on the extract of the material. In both cases, the morphology was similar to the negative control. Figures 4(a)–4(c) and Figures 5(a)–6(c) show the optical micrographs of direct contact test and test on extract respectively.

### 3.5. *In Vitro* Cell Adhesion Studies

**Figure 7:**
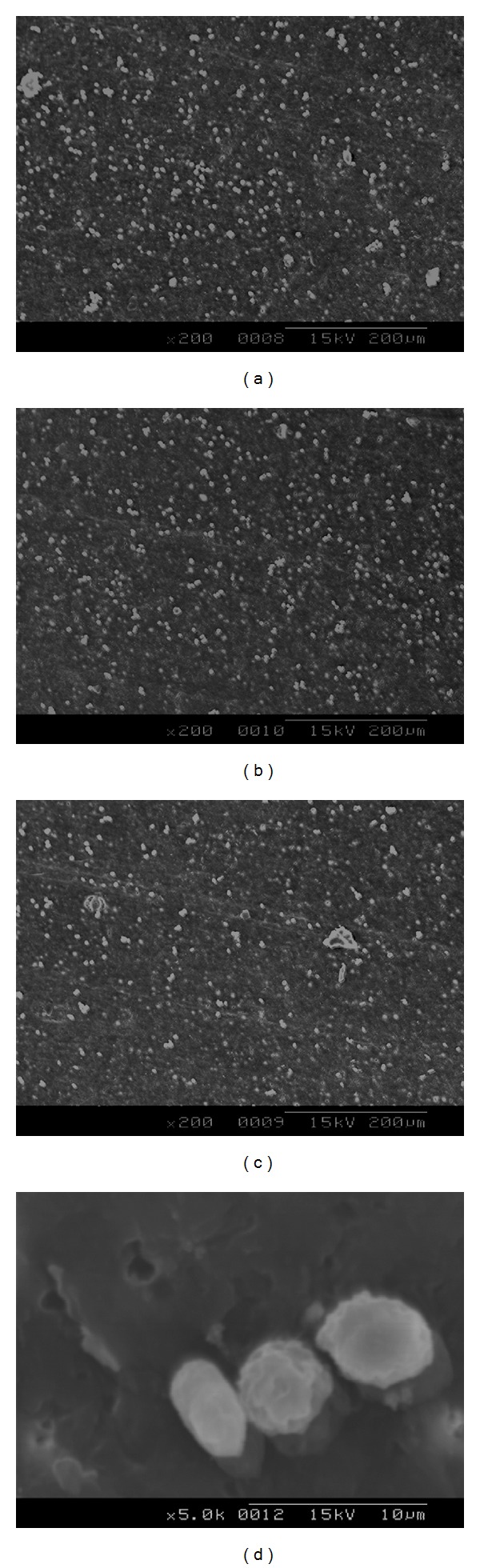
SEM micrographs showing the cell growth over the surface of blend samples with microporosity on surface.

Figures 6 and 7 show the SEM micrographs of cells proliferated on blend samples surfaces without and with surface microporosity. It can be observed that the fibroblast cells proliferated onto the surface of the membranes exhibited a flattened morphology that demonstrated a good adherence to the surface. In contrast, in blend samples without surface microporosity, the fibroblasts exhibited round morphology, showing low cell adherence and proliferation. It is well known that to mimic the topological and microstructure characteristics of the extracellular matrix, a biomaterial surface must have high degree of porosity, high surface-to-volume ratio, and high degree of pore interconnection, appropriate pore size, and geometry control [18]. The increase in cell proliferation on samples with microporosity suggests that the porosity of the surface helped the cells to adhere closely to the samples.

More information regarding the proliferation and growth of cell lines over the blend surface can be gathered by quantitative studies such as cell counting kit-8 (CCK-8) cell proliferation assay, which will be included in the future prospects of the present investigation.

## 4. Conclusions

Compatibilized blend of TPU and PDMS was prepared by reactive blending technique and microporosity was created on the surface of this blend material. *In vitro* cytotoxicity studies indicate that the material is noncytotoxic towards L929 cell lines. The surface microporosity favoured the ingrowth and proliferation of the cells as evidenced from scanning electron micrographs. The blend material also possesses sufficient strength to prevent cutting and tearing through suture holes. This material could be further developed for various biomedical applications.

## Figures and Tables

**Table 1:** Suture holding strength measurement results for TPU : PDMS blends in the ratio 80 : 20 with compatibilizer content from 0–5 pbw.

Sample code	Max. load (*N*)	Max. strain (%)
80 : 20	10.2 (9.7)	86 (87)
80 : 20 : 2	11.6 (11.1)	62 (68)
80 : 20 : 5	9.7 (8.7)	67 (64)

The values given in bracket are for samples with surface microporosity.
